# MCAM interacts with integrin β1 to promote EGFR-TKI resistance in lung adenocarcinoma through the JAK3 signalling pathway

**DOI:** 10.1186/s12967-025-06874-9

**Published:** 2025-07-25

**Authors:** Saiqun Zhang, Yuling Chen, Chang Li, Jian Zhao, Min Jiao, Mengzhu Zhang, Chenkang Ma, Anqi Wang, Jianjun Li, Yuanyuan Zeng, Jianjie Zhu, Chuanyong Mu, Jian-an Huang, Zeyi Liu

**Affiliations:** 1 Department of Pulmonary and Critical Care Medicine, The First Affiliat ed Hospital of Soochow University, Suzhou, 215006 China; 2 Institute of Respiratory Diseases, Soochow University, Suzhou, 215006 China; 3 Suzhou Key Laboratory for Respiratory Diseases, Suzhou, 215006 China; 4 Cancer Institute, Suzhou Medical College, Soochow University, Suzhou, 215123 China

**Keywords:** EGFR-TKIs, MCAM, Integrin β1, JAK3, Resistance

## Abstract

**Background:**

Epidermal growth factor receptor (EGFR) tyrosine kinase inhibitors (TKIs) have exhibited remarkable efficacy against EGFR-mutated lung cancer. However, the majority of patients receiving EGFR-TKIs face a grim prognosis due to acquired drug resistance. Melanoma cell adhesion molecule (MCAM), a highly glycosylated type I transmembrane protein belonging to the immunoglobulin superfamily, is upregulated in EGFR-TKI-resistant EGFR-mutant lung adenocarcinoma. The objective of this study was to investigate the role and mechanism of MCAM in mediating resistance to EGFR-TKIs.

**Methods:**

qRT-PCR, Western blot, and immunofluorescence techniques were employed to analyze the expression of MCAM in EGFR-TKI sensitive and resistant cells.CCK-8 assay and subcutaneous xenograft model in nude mice were utilized to investigate the correlation between MCAM expression level and resistance to EGFR-TKIs. Furthermore, receptor tyrosine kinase (RTK) phosphorylation antibody array, co-immunoprecipitation, immunofluorescence, and dual-luciferase assay were employed to elucidate its molecular mechanism.

**Results:**

The expression level of MCAM was significantly higher in EGFR-TKI resistant lung adenocarcinoma cells compared to EGFR-TKI sensitive lung adenocarcinoma cells. CCK-8 cytotoxicity assay demonstrated that overexpression of MCAM enhanced resistance to EGFR-TKIs in EGFR-mutant lung adenocarcinoma cells. Overexpression of MCAM promoted the growth of transplanted tumors in nude mice, while having a limited effect on the efficacy of EGFR-TKIs. Moreover, interaction between MCAM and integrin β1 activated the downstream JAK3 signaling pathway, thereby promoting resistance to EGFR-TKIs. The transcription factor STAT2 activates transcription of the target gene MCAM by binding to its promoter region.

**Conclusions:**

MCAM can enhance the resistance of EGFR-mutant lung adenocarcinoma cells to EGFR-TKIs, both in vitro and in vivo. Through its interaction with integrin β1, MCAM activates JAK3 protein, thereby stimulating cell proliferation and contributing to the development of acquired resistance in sensitive cell lines. The expression of MCAM is regulated by the transcription factor STAT2. This study presents a novel therapeutic strategy for patients with EGFR-TKI resistance.

**Supplementary Information:**

The online version contains supplementary material available at 10.1186/s12967-025-06874-9.

## Introduction

Cancer is one of the most serious health problems in the world, and lung cancer is the leading cause of death from cancer. According to the latest statistics, the expected 5-year survival rate of lung cancer patients is only 25%, and lung cancer is still the main cause of cancer-related death worldwide [1]. Epidermal growth factor receptor tyrosine kinase inhibitors (EGFR-TKIs) have a significant curative effect on EGFR-mutant lung cancer; however, patients treated with EGFR-TKIs eventually experience disease progression due to acquired drug resistance [2, 3]. The mechanisms of acquired resistance to TKIs are due to secondary mutations of EGFR, mutations in genes encoding proteins downstream of EGFR, and the bypass of activation or phenotypic changes in signalling pathways [4–6]. Therefore, further exploration of the mechanism of EGFR-TKI resistance, which is highly important for overcoming or delaying resistance to EGFR-TKIs and improving the prognosis of patients, is urgently needed.

Melanoma cell adhesion molecule (MCAM) belongs to the immunoglobulin superfamily and is a transmembrane glycoprotein. Under pathological conditions such as inflammation and tumorigenesis, MCAM is upregulated in related cells and is considered a reliable marker for many types of cancer, and MCAM overexpression is associated with the initial development or metastasis of most cancers [7]. In addition to the membrane-anchored form of MCAM, a soluble form is produced by the shedding of the membrane-associated form, which is secreted by tumours expressing MCAM and has autocrine effects on cancer cell proliferation and survival. The plasma soluble MCAM levels of NSCLC patients are significantly higher than those of healthy subjects, and are significantly associated with shorter progression-free survival and overall survival [8]; however, the role of MCAM in lung adenocarcinoma patients with EGFR mutations still remains to be explored. Therefore, this study explored the expression and function of MCAM in EGFR-mutant the lung adenocarcinoma and the specific mechanism of EGFR-TKI resistance.

This study aimed to identify whether the expression of MCAM correlates with acquired resistance, as well as the exact signalling pathways involved in MCAM-mediated acquired resistance to EGFR-TKIs. The study hypothesizes that MCAM promotes resistance via Integrin β1-JAK3 activation.

## Materials and methods

### Cell culture

HCC827 (RRID: CVCL_2063) and HEK293T (RRID: CVCL_0063) cells were acquired from the Chinese Academy of Sciences (Shanghai, China). Gefitinib-resistant HCC827 cells (HCC827GR) and osimertinib-resistant HCC827cells (HCC827OR) were constructed in the laboratory. The cell lines were subjected to regular short tandem repeat identification for authentication and the detection of mycoplasma contamination. The cells were cultured in RPMI 1640 or DMEM containing 10% foetal bovine serum (Gibco, Carlsbad, CA, USA) and L-glutamine (Invitrogen, Carlsbad, CA, USA) at 37 °C with 5% CO_2_.

### CCK-8 cell viability assay

The viability of NSCLC cells seeded at a density of 3000 cells per well in 96-well plates was assessed using the CCK-8 assay. Culture medium supplemented with foetal bovine serum (10%) was added to each well. Following a 24-hour incubation, the cells were exposed to varying concentrations of gefitinib (APExBIO, USA) and osimertinib (APExBIO, USA) for an additional 72 h. Thereafter, the absorbance at 450 nm was determined to quantify cell proliferation.

### RNA interference

Prior design and synthesis of short interfering RNA (siRNA) sequences targeting MCAM or STAT2 were conducted by GenePharma (Suzhou, China). The target sequences are detailed in Table S1 of Attached File 1. A noninterfering siRNA served as the negative control. The cells were transiently transfected with 100 pmol of siRNA using Lipofectamine 2000 (Invitrogen, USA), and subsequent experiments were conducted at 48 h post transfection.

### RNA isolation and quantitative real-time PCR assays

Purified total RNA was extracted from NSCLC using an RNAiso Plus kit (Takara, Kusatsu, Shiga, Japan). The RNA samples were subsequently solubilized in diethyl pyrocarbonate-treated water and preserved at -80 °C for subsequent analysis. cDNA was synthesized using the M-MLV First Strand Synthesis Kit (Life Technologies, Carlsbad, CA, USA). The primers used were procured from Sangon Biotechnology (Shanghai, China). The cDNA products were assayed via 2x SYBR-Green qPCR SuperMix (High ROX) on an ABI StepOne Plus real-time PCR machine (Applied Biosystems, Foster City, CA, USA) according to the manufacturer’s protocols. The experiments were replicated three times to ensure reproducibility. The sequences of the gene-specific primers are provided in Additional File 2: Table S2. Gene expression was quantified using the ΔΔCt method, with β-actin serving as the housekeeping gene for normalization.

### Lentiviral production and transduction

Lentiviruses were produced by transfecting 293T packaging cells with the lentiviral envelope plasmid pMD2G and the packaging plasmid psPAX2. The empty vector served as the negative control. The lentiviral supernatants were collected 48 h after transfection. HCC827 cells were seeded in six-well plates and transduced with lentiviruses plus polybrene (Beyotime Biotechnology, China). The transduced cells were selected with 2 µg/ml puromycin (Amresco, Solon, OH, USA). MCAM expression was evaluated by Western blotting after selection.

### Western blot analysis

Protein lysates were isolated from NSCLC cells using RIPA buffer (Cell Signaling Technology), followed by centrifugation at 12,000 RPM for 15 min. The supernatant was then collected and incubated at 4 °C. The protein samples were subsequently aliquoted and stored at -80 °C. For subsequent analysis, the protein lysates were subjected to sodium dodecyl sulfate‒polyacrylamide gel electrophoresis (SDS‒PAGE). After electrophoresis, the separated proteins were transferred to nitrocellulose membranes (Millipore) via a semidry transfer system. The membranes were subsequently incubated in a blocking solution containing 5% BSA in TBST (Tris-buffered saline with Tween 20) for 1 h at room temperature, followed by probing with specific primary antibodies overnight at 4 °C. The following day, the membranes were washed and reprobed with the corresponding secondary antibodies at 15–25 °C for 2 h. The protein bands were visualized using an enhanced chemiluminescence (ECL) detection kit (Thermo Fisher) according to the manufacturer’s protocol. β-Actin was used as the loading control. Anti-MCAM, anti-Integrin β1 (RRID: AB_11178800), anti-pAKT (Ser473 D9E) (RRID: AB_2315049) and anti-AKT (RRID: AB_329827) antibodies from Cell Signaling Technology were used. Anti-pJAK3 and anti-JAK3 (RRID: AB_775811) antibodies from Abcam. Anti-pSTAT2 antibodies from Absin were used. Anti-β-Actin (RRID: AB_2687938) and anti-STAT2 (RRID: AB_10644445) antibody was obtained from Proteintech.

### Co-immunoprecipitation

Co-immunoprecipitation was performed with HCC827 cells. The procedure involved incubating the cells with magnetic beads conjugated with FLAG (SELLECK, USA) and IgG at 4 °C overnight. The supernatant was subsequently separated from the beads by magnetic separation the following day. Then, 1 mL of immunoprecipitation buffer was added to the beads, and the mixture was centrifuged at high speed three times to obtain a clean pellet of magnetic beads. Finally, the beads were incubated in 2x protein loading buffer at 100 °C for 10 min. The FLAG- and IgG-bound proteins were then separated via SDS‒PAGE and analysed by Western blotting.

### Immunofluorescence staining

The cells were seeded into 12-well plates pre-equipped with glass slides. Following a 24-hour incubation, the cells were gently washed with phosphate-buffered saline (PBS) and reached a confluence of 40–50%. The cells were subsequently fixed with 4% paraformaldehyde for 30 min, followed by permeabilization with a 0.5% solution of Triton X-100 for an additional 20 min. Prior to antibody labelling, a 5% solution of bovine serum albumin was used as a blocking agent. The primary antibodies, including an anti-MCAM antibody (CST, USA) and an anti-integrin β1 antibody (Abcam, USA, RRID: AB_775726), were applied sequentially, followed by an incubation with the corresponding secondary antibodies conjugated withCy3 and FITC.

### Luciferase reporter assay

MCAM promoter sequences were cloned and inserted into pGL3 luciferase reporter vectors (Genecreate, China). The pGL3 basic vector was used as a negative control. The cells were plated on 24-well plates at 50,000 cells per well and allowed to adhere overnight. The next day, the cells were transfected with luciferase reporters and incubated at 37 °C for 48 h. The cell extracts were assayed as recommended by the manufacturer (Vazyme, China).

### In vivo xenograft assays

In the CDX study, 5 × 10^6^ tumour cells suspended in 100 µl of PBS were subcutaneously injected into athymic nu/nu BALB/c mice at approximately 5 weeks of age. The tumour volume was measured every 2 to 3 days using digital callipers and calculated according to the formula V = length×width^2^ × 0.5. When the tumour volume reached approximately 150 mm³, Mice were randomly assigned to different treatment groups, with five mice per group. The mice were euthanized when the endpoint of the experiment was reached or when the tumour volume exceeded 1000 mm³. The tumours were subsequently excised, weighed, and promptly frozen in liquid nitrogen for subsequent analysis. The mice were housed in ventilated cages at the Laboratory Animal Center of Soochow University (Suzhou, China). The mice were maintained in a pathogen-free environment with a temperature ranging from 22 °C to 26 °C and a 12-hour light/dark cycle. Sterile pellet food and water were provided ad libitum. All procedures and experiments involving animal studies were conducted in accordance with the approval of the Animal Care and Use Committee (IACUC) and conformed to the guidelines for animal care and use at the Soochow University School of Medicine.

### Statistical analysis

The experiments were repeated three times. Statistical analysis was performed using two-tailed Student’s t test or analysis of variance (ANOVA) followed by Tukey’s multiple comparisons test. The results are presented as the means ± SDs. Statistical analysis was performed with GraphPad Prism 7.0 (GraphPad, CA, USA). *P* < 0.05 was considered significant.

## Results

### MCAM is highly expressed in EGFR-TKI-resistant lung adenocarcinoma cells

**Fig. 1:**
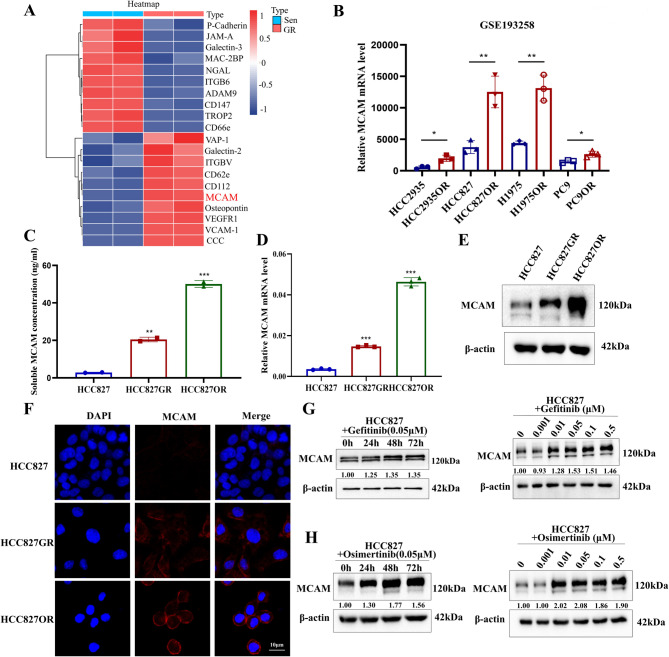
MCAM is highly expressed in EGFR-TKI-resistant lung adenocarcinoma cells. (**A**) The heatmap shows the MCAM expression level in the HCC827 and HCC827GR soluble receptor array chips. (**B**) MCAM expression in HCC2935, HCC827, H1975, and PC9 osimertinib-resistant cells was assessed using the GSE193258 dataset. (**C**) The expression of secreted MCAM (sOPN) was verified by ELISA in HCC827GR and HCC827OR cells. (**D-E**) The expression levels of MCAM in HCC827, HCC827GR, and HCC827OR cells were assessed using qRT‒PCR (**D**) and Western blotting (**E**). (**F**) Immunofluorescence staining was used to detect the MCAM expression level. (**G-H**) Western blot analysis was conducted to assess the expression of MCAM in HCC827 cells treated with gefitinib and osimertinib for various times or with various concentrations.*P < 0.05; **P < 0.01; ***P < 0.001

First, based on previous data from HCC827 and HCC827GR cells from a high-flux membrane protein chip array [9]and the GEO public database (https://www.ncbi.nlm.nih.gov/geo/), MCAM expression was increased in multiple EGFR-TKI-resistant cell lines (Fig. 1A, B; Fig. S1A-B). We also detected the expression of secreted MCAM (sMCAM) via ELISA. sMCAM expression was apparently higher in HCC827GR and HCC827OR cells than in HCC827 cells (Fig. 1C). MCAM mRNA and protein expression levels in HCC827 EGFR-TKI-resistant cells were significantly higher than those in EGFR-TKI-sensitive cells according to qRT‒PCR, Western blotting and immunofluorescence staining (Fig. 1D-F).

HCC827 cells were cultured and exposed to 0.05 µmol/L gefitinib or osimertinib to investigate a potential secondary increase in MCAM expression in lung adenocarcinoma cells. qRT‒PCR and Western blotting were used to detect the expression levels of MCAM at different time points. MCAM expression increased with increasing EGFR-TKI treatment time, and results obtained with the concentration gradient of gefitinib and osimertinib also confirmed this result (Fig. 1G-H; Fig. S1C-F).

### MCAM mediated EGFR-TKI resistance in lung adenocarcinoma cancer cells in vitro

**Fig. 2:**
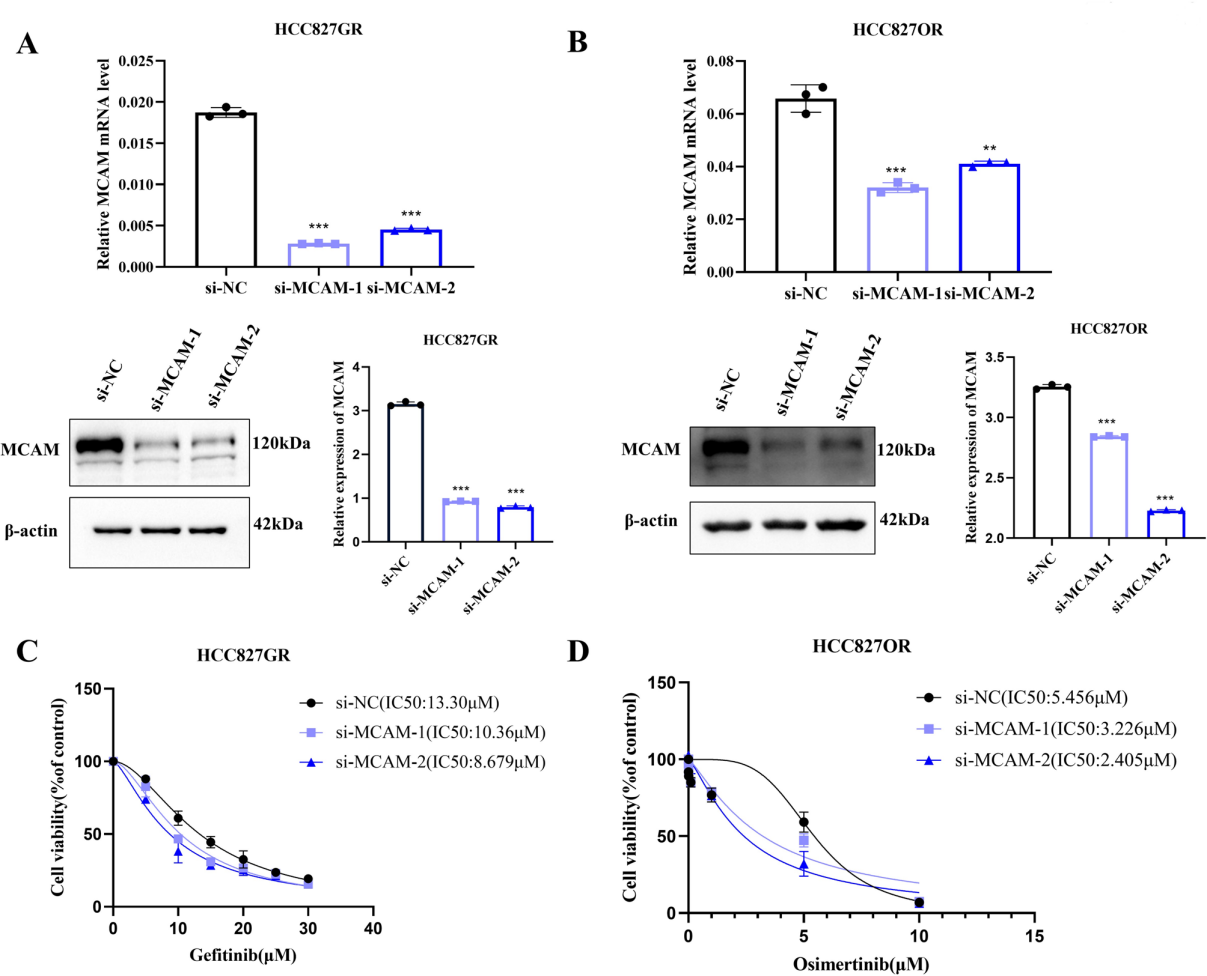
MCAM mediated EGFR-TKI resistance in lung adenocarcinoma cancer cells in vitro. (**A-B**) qRT‑PCR and Western blotting were performed to detect the expression of MCAM in HCC827GR and HCC827OR cells after transfection with si‑MCAM. (**C**) The sensitivity of HCC827GR cells transfected with si‑MCAM or si‑NC to gefitinib was determined via CCK‑8 assays. (**D**) The sensitivity of HCC827OR cells transfected with si‑MCAM or si‑NC to osimertinib was determined via CCK‑8 assays.**P < 0.01; ***P < 0.001

A CCK-8 cytotoxicity assay was used to detect the effect of MCAM interference on EGFR-TKI resistance in HCC827GR and HCC827OR cells in vitro. After MCAM knockdown (Fig. 2A-B), HCC827GR and HCC827OR cells were treated with different concentrations of gefitinib and osimertinib, and the half maximal inhibitory concentration (IC50) was calculated. The results showed that the IC50 of gefitinib or osimertinib decreased after MCAM knockdown in EGFR-TKI-resistant lung adenocarcinoma cells (Fig. 2C-D), indicating that the sensitivity to EGFR-TKIs was restored to a certain extent. Ectopic MCAM expression in HCC827 cells was examined after treatment with the IC50 of gefitinib or osimertinib to further clarify whether MCAM is involved in the acquired resistance of lung adenocarcinoma cells. These results suggested that the overexpression of MCAM significantly increased the resistance of sensitive cells to gefitinib or osimertinib (Fig. S2A-C).

### MCAM overexpression induces tumour resistance to EGFR-TKIs in vivo

**Fig. 3:**
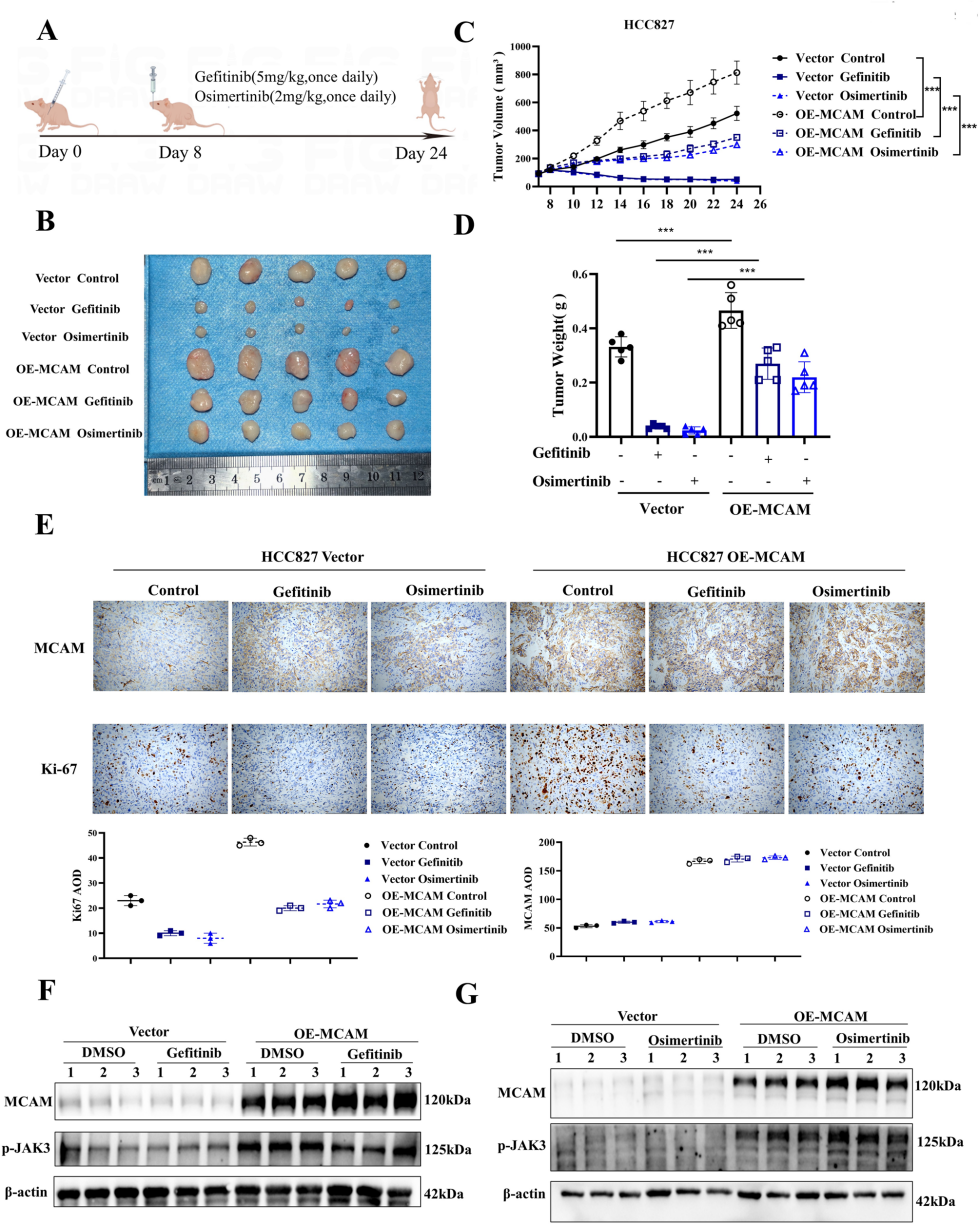
MCAM overexpression induces tumour resistance to EGFR-TKIs in vivo. (**A**) Schematic illustration of the animal experiments. (**B-D**) Growth of HCC827 MCAM-CDX tumours and HCC827 vector-CDX tumours treated with gefitinib and osimertinib (**B-C**) and changes in tumour weight (**D**). The CDX-bearing mice (*n* = 5 mice per group) were treated with vehicle (1% sodium carboxymethyl cellulose, once daily), gefitinib (5 mg/kg, once daily), or osimertinib (2 mg/kg, once daily) by oral gavage. (**E**) IHC staining for Ki-67 for an in situ analysis of cell proliferation. Ki-67 is a cell proliferation marker. (**F-G**) The effects of gefitinib and osimertinib treatment on MCAM expression in HCC827-CDX tumours were assayed by Western blotting. Three randomly selected tumour samples per group are shown. scale bars = 200 μm.,***P < 0.001

The above experiments confirmed that MCAM was involved in the resistance of lung adenocarcinoma cells to EGFR-TKIs in vitro. Four-week-old BALB/c nude mice were randomly divided into a vector group and an MCAM overexpression group, with 15 nude mice in each group, to further verify whether MCAM plays a role in vivo. The nude mice were subcutaneously inoculated with vector- or MCAM-overexpressing cells, tumour formation was observed, and the tumour volume was measured every other day. When the tumour volume reached approximately 100 mm^3^, the vector- and MCAM-overexpressing groups were randomly divided into a solvent control group, a gefitinib group and a osimertinib group. Medicine was provided by gavage every day (Fig. 3A), the volumes of tumours were measured every other day and tumour growth curves were drawn. After treatment and euthanasia of the groups of nude mice, tumours were removed, photographed and weighed (Fig. 3B). As shown in the tumour growth curve (Fig. 3C) and graph of tumour weight (Fig. 3D), the tumour volume and weight in the MCAM-overexpressing group were significantly greater than those in the control group, and the tumour volume and weight in the control group were significantly lower after treatment with gefitinib or osimertinib, indicating that the tumours responded to gefitinib or osimertinib treatment. The treatment sensitivity of the MCAM-overexpressing group was much lower than that of the control group. In addition, Ki-67 immunohistochemical staining revealed that gefitinib and osimertinib were less effective at inhibiting tumour cell proliferation in the MCAM-overexpressing group than in the control group (Fig. 3E). Moreover, continuous EGFR-TKI treatment induced the upregulation of MCAM expression in tumours (Fig. 3F-G).

### MCAM activates the JAK3 signalling pathway in lung adenocarcinoma

**Fig. 4:**
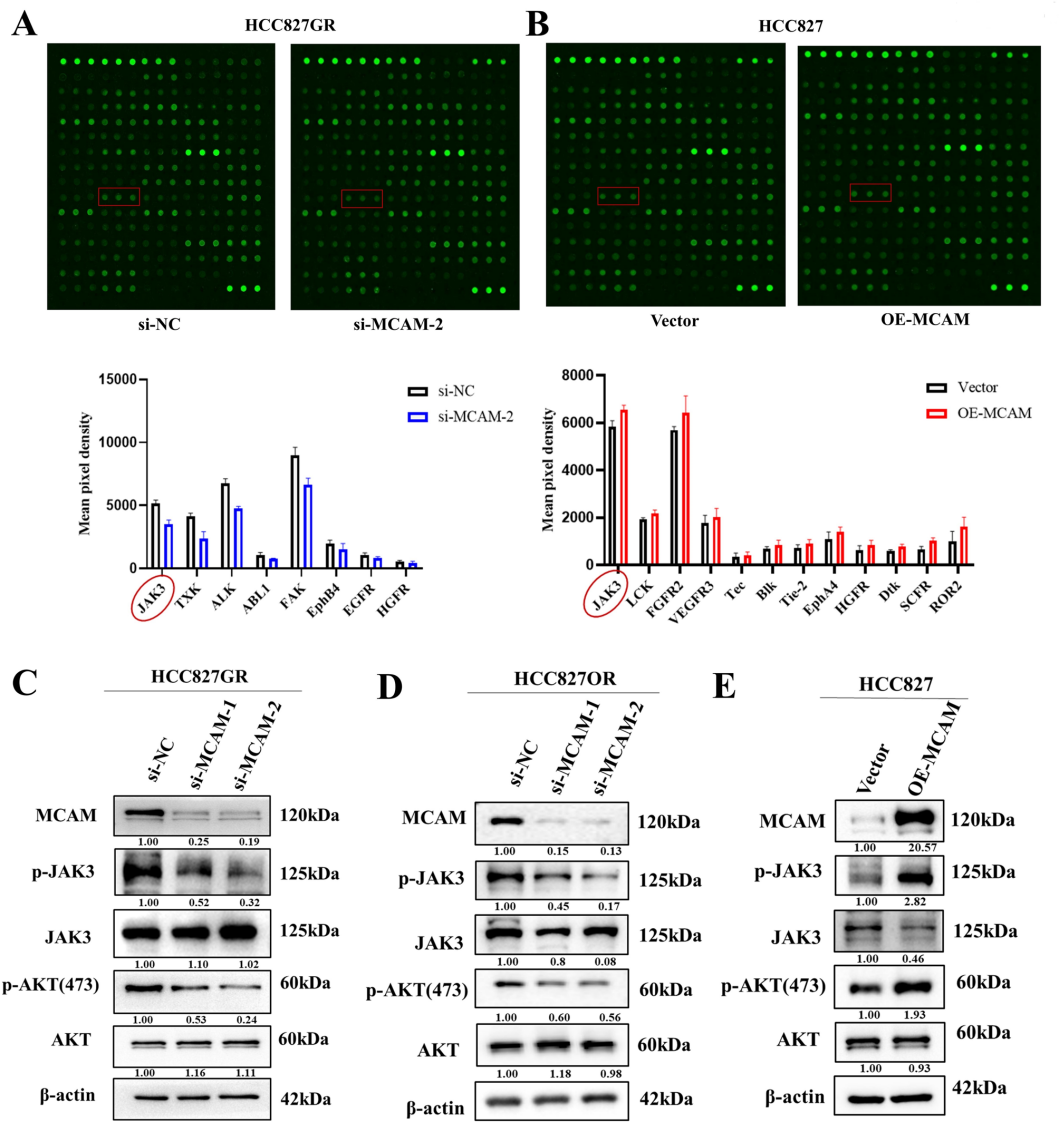
MCAM activated the JAK3 signalling pathway in lung adenocarcinoma. (**A**) An RTK array was used to identify the proteins whose expression was downregulated in HCC827GR cells upon MCAM interference, as well as the proteins whose expression was upregulated in HCC827 cells overexpressing MCAM. (**B-C**) Western blotting was used to detect the phosphorylation levels of JAK3 and downstream AKT in the HCC827GR (**B**) and HCC827OR (**C**) knockdown groups and the NC group. D, Western blotting was used to detect changes in the phosphorylation levels of JAK3 and AKT in the HCC827-overexpressing MCAM group and the vector group

In this study, HCC827GR cells in the si-NC group and si-MCAM interference group, as well as HCC827 cells in the vector group and MCAM-overexpressing group, were analysed via a human RTK phosphorylation array. The results revealed that the level of Janus tyrosine kinase 3 (JAK3) phosphorylation decreased after MCAM knockdown but increased after MCAM overexpression, suggesting that MCAM exerted its effect by activating the JAK3 signalling pathway (Fig. 4A-B). Then, Western blotting was performed to detect changes in the level of JAK3 phosphorylation in the control group and the MCAM-overexpressing group, and the phosphorylation level of AKT, which is downstream of JAK3, was detected. The results revealed that in the MCAM-overexpressing group, JAK3/AKT phosphorylation levels increased significantly. Conversely, when MCAM was knocked down, the level of phosphorylated JAK3 decreased, and the level of phosphorylated AKT decreased (Fig. 4C-E), further confirming that MCAM can activate the JAK3 signalling pathway.

### MCAM promotes cellular resistance by activating JAK3 through an interaction with Integrin β1

**Fig. 5:**
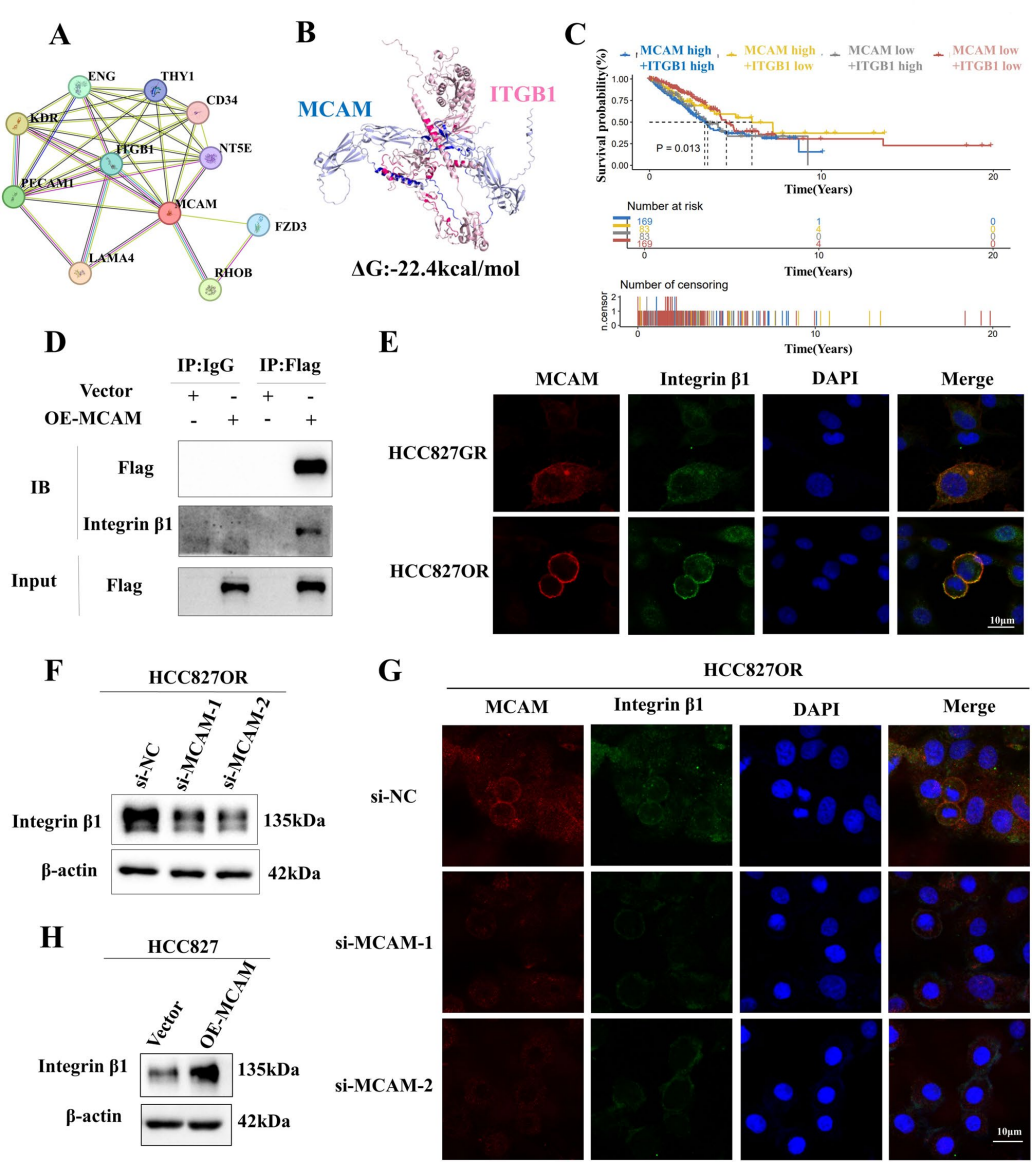
MCAM interacted with Integrin β1. (**A**) MCAM-interacting proteins were predicted via the STRING database. (**B**) Molecular dynamics and molecular docking between MCAM and Integrin β1 were predicted using the ZDOCK website. (**C**) The survival analysis demonstrated variations in the survival rate based on the expression status of MCAM and Integrin β1. (**D**) The interaction between MCAM and Integrin β1 was detected via coimmunoprecipitation.(**E**) Immunofluorescence detection of the colocalization of MCAM and Integrin β1. (**F**) Integrin β1 expression was detected by Western blotting following the knockdown of MCAM. (**G**) Integrin β1 expression in HCC827OR cells was detected by immunofluorescence staining following MCAM knockdown. (**H**) Western blotting was used to detect the expression of Integrin β1 after overexpression of MCAM. scale bars = 10 μm

**Fig. 6:**
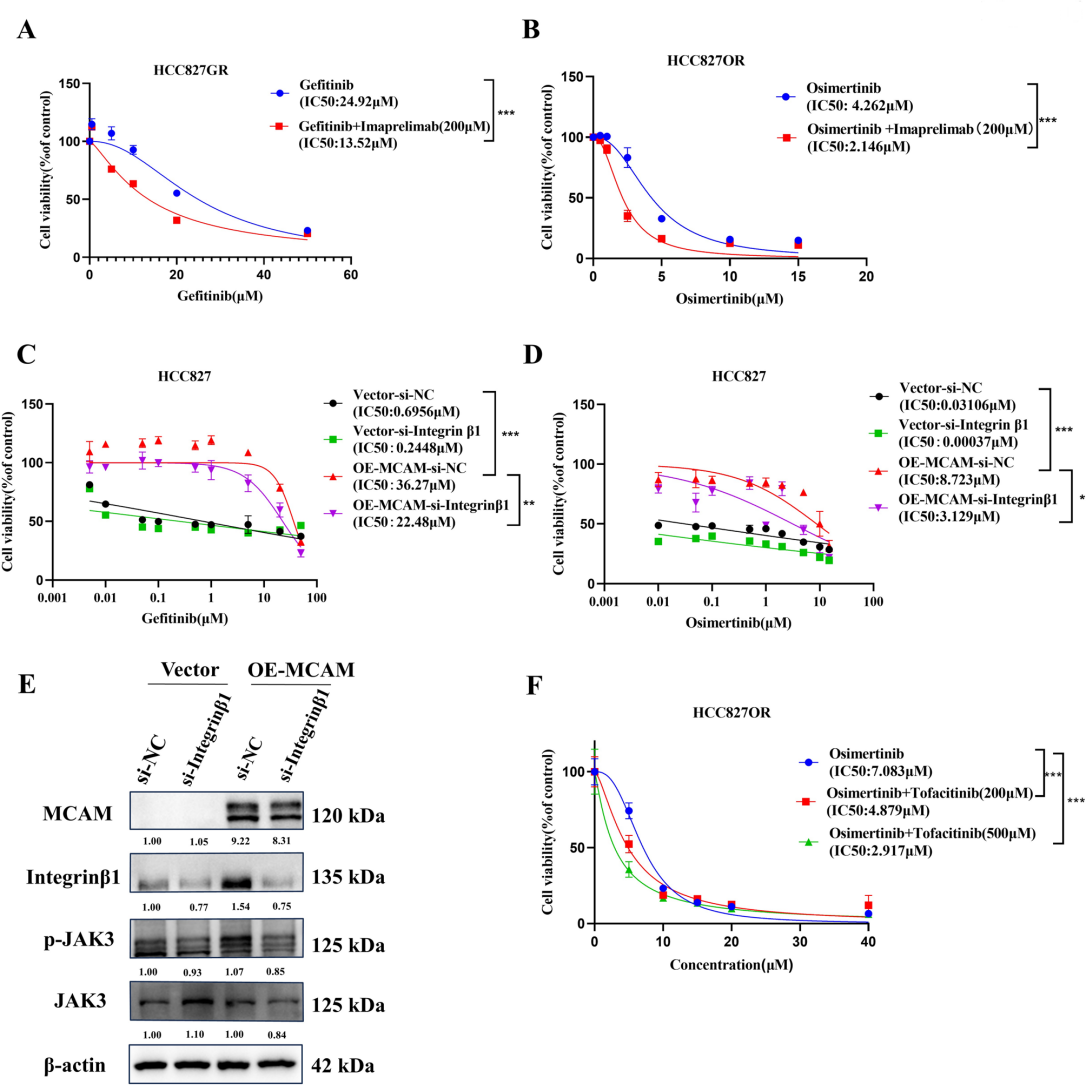
MCAM promotes cell resistance by activating JAK3 via an interaction with Integrin β1. (**A**) The sensitivity of imaprelimab-treated HCC827GR cells to gefitinib was determined via CCK-8 assays. Imaprelimab is a humanized monoclonal antibody against MCAM. (**B**) The sensitivity of imaprelimab-treated HCC827OR cells to osimertinib was determined via CCK-8 assays. (**C**-**D**) Knockdown of Integrin β1 inhibits MCAM-induced cell resistance. (**E**) Knockdown of Integrin β1 inhibits MCAM-induced activation of the JAK3 signalling pathway in HCC827 cells. (**F**)Treatment of HCC827OR cells with osimertinib and tofacitinib for 72 h before determining cell viability with a CCK8 assay. *P < 0.05; **P < 0.01; ***P < 0.001

Based on the above results, we used the STRING database (https://cn.string-db.org/) to predict possible proteins interacting with the MCAM protein as a method to determine how MCAM affects the JAK3 signalling pathway (Fig. 5A). MCAM and Integrin β1 form a complex leading to radio resistance in triple-negative breast cancer [10]. Immunoprecipitation coupled with mass spectrometry (IP/MS) was employed to identify the putative co-receptors of MCAM in MCAM-overexpressing HCC827 cells. IP/MS reveals the protein network involved in MCAM interactions, encompassing integrin α6 and integrin α4 within the integrin family. Subsequently, we integrated our mass spectrometry findings from resistant strains and observed that only a6 exhibited elevated levels in these strains (Fig. S2D). Furthermore, it has been demonstrated that integrin α6 forms complexes with integrin β1 [11], and we discovered a positive correlation between MCAM and integrin β1(Fig. S2E-F). Then ZDOCK (https://zdock.umassmed.edu/) was used for both structures in the docking simulation, and the molecular docking results suggested that MCAM can interact with Integrin β1 (Fig. 5B). The results of the survival analysis indicate that high expression levels of MCAM and Integrin β1 are associated with a lower overall survival rate (Fig. 5C). Co-immunoprecipitation experiments were subsequently performed to verify the interaction between MCAM and Integrin β1, and the results revealed that MCAM and Integrin β1 interacted with each other (Fig. 5D). Immunofluorescence experiments revealed that in HCC827GR and HCC827OR cells, both MCAM and Integrin β1 were located in the cell membrane and were colocalized (Fig. 5E). Furthermore, Western blot analysis verified that the overexpression of MCAM increased the expression of Integrin β1, and the knockdown of MCAM reduced the expression of Integrin β1 (Fig. 5F, H; Fig. S2G), which was confirmed by immunofluorescence staining (Fig. 5G; Fig. S2H). These results suggest that MCAM may activate JAK3 via Integrin β1. We subsequently transfected an Integrin β1 siRNA into MCAM-overexpressing and control cells. The Western blot results revealed that MCAM overexpression increased the expression of the Integrin β1 and p-JAK3 proteins. Knockdown of Integrin β1 in MCAM-overexpressing cell lines reduced the expression of both the aberrantly activated Integrin β1 and p-JAK3 proteins (Fig. 6E). A CCK-8 cytotoxicity assay also revealed that reducing Integrin β1 expression could partially rescue the resistance to EGFR-TKIs caused by MCAM overexpression (Fig. 6C-D).

The MCAM monoclonal antibody was introduced in this study to further validate the role of MCAM in mediating drug resistance. Through the CCK-8 cytotoxicity assay, we observed a decrease in the IC50 of drug-resistant cells when gefitinib or osimertinib was combined with imaprelimab (Fig. 6A-B). Furthermore, this study examined the alterations in drug resistance when the JAK3 inhibitor tofacitinib was used in combination with osimertinib. The findings demonstrated that the co-administration of the JAK3 inhibitor and osimertinib markedly reversed cellular resistance to osimertinib, with higher concentrations of the JAK3 inhibitor exhibiting a more pronounced synergistic effect (Fig. 6F). In conclusion, MCAM induced EGFR-TKI resistance in lung adenocarcinoma by interacting with Integrin β1 and then activating the JAK3 signalling pathway.

### STAT2 induces MCAM transcription

**Fig. 7:**
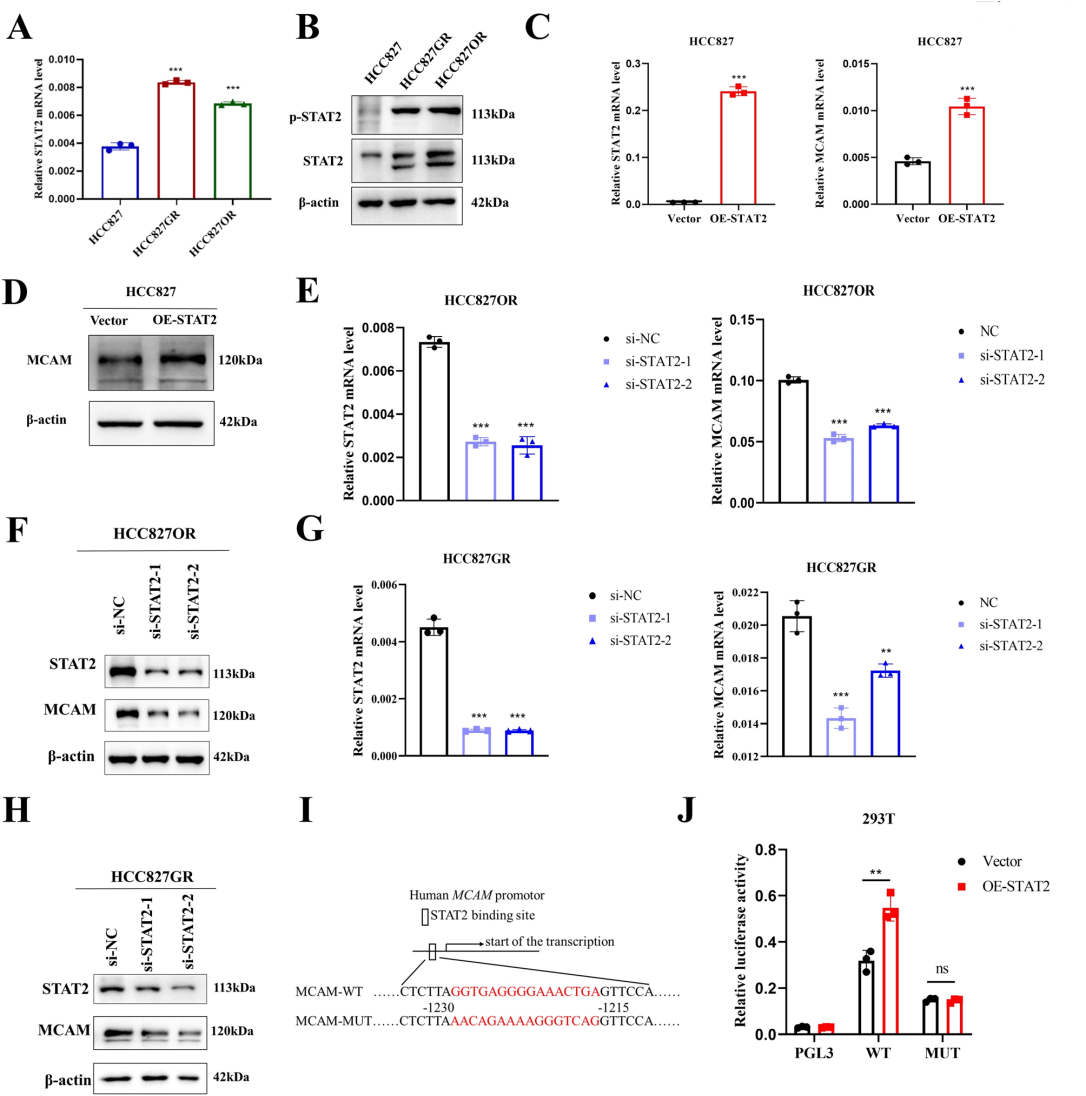
STAT2 induces MCAM transcription. (**A-B**) qRT‒PCR and Western blotting were performed to detect STAT2 expression levels in HCC827, HCC827GR and HCC827OR cells. (**C-D**) After STAT2 overexpression in HCC827 cells, qRT‒PCR (**C**) and Western blotting (**D**) were performed to detect MCAM expression levels. (**E-F**) After interfering with STAT2 in HCC827OR cells, MCAM expression levels were determined by qRT‒PCR (**E**) and Western blotting (**F**). (**G-H**) After interfering with STAT2 in HCC827GR cells, MCAM expression levels were determined by qRT‒PCR (**G**) and Western blotting (**H**). (**I**) Design of the MCAM wild-type and mutant plasmids. (**J**) Changes in luciferase activity were assessed using the dual-luciferase assay. **P < 0.01; ***P < 0.001

According to the above results, the mRNA level of MCAM was increased in EGFR-TKI-resistant cell lines compared with EGFR-TKI-sensitive cell lines. Therefore, here, we investigated the cause of the increase in MCAM expression. The hTFtarget transcription factor database (https://guolab.wchscu.cn/hTFtarget) and GTRD (http://gtrd.biouml.org) were used to predict transcription factors regulating MCAM expression. Furthermore, according to the GEO database (GSE193258), genes whose expression was not significantly increased in EGFR-TKI-resistant cell lines compared with sensitive cell lines were excluded, and 6 alternative transcription factors were obtained: RORA, FOXO4, STAT2, BACH2, MEIS3, and ETV7 (Fig. S3A). Since the above RTK results suggest that the JAK3 signalling pathway is activated and because the JAK/STAT signalling pathway is considered one of the central communication nodes in cellular functions, we examined whether the expression of Signal transducer and activator of transcription 2 (STAT2) was secondarily increased in EGFR-TKI-resistant lung adenocarcinoma. The mRNA and protein levels of STAT2 were significantly higher than those in sensitive lung adenocarcinoma cells (Fig. 7A-B). After treatment with varying concentrations of gefitinib and osimertinib, we observed a gradual increase in the expression levels of STAT2 and p-STAT2. Additionally, the results of nucleoplasmic dissociation indicated the increased level of p-STAT2 in both the cytoplasm and nucleus (Fig. S3B-D). HCC827 cells were subsequently transfected with a STAT2 overexpression plasmid, and the results revealed that the mRNA and protein expression levels of MCAM were significantly increased after the overexpression of STAT2 (Fig. 7C-D). The qRT‒PCR and Western blot results revealed that the knockdown of STAT2 downregulated the expression of MCAM (Fig. 7E‒H). These results suggest that STAT2 transcriptionally regulates MCAM gene expression. We subsequently used the JASPAR database (http://jaspar.genereg.net/) to predict the potential binding sites for STAT2 in the MCAM promoter region, and constructs containing the wild-type MCAM binding sites and the mutant binding sites were inserted into recombinant plasmids (Fig. 7I). After cotransfecting 293T cells with an overexpression plasmid for STAT2 and a wild-type plasmid for MCAM, we observed a significant increase in luciferase reporter gene activity. However, when the binding site was mutated and STAT2 was overexpressed, no significant change in the luciferase activity of the mutant MCAM plasmid was detected (Fig. 7J). These findings indicate that MCAM is a target gene of STAT2.

## Discussion

Targeted therapeutic regimens are routinely recommended for the treatment of NSCLC patients with corresponding gene mutations [12]. EGFR-TKIs are preferentially utilized for the treatment of sensitive EGFR mutations, including EGFR exon 19 deletion (Ex19del) and the L858R mutation [13]. With the development and clinical application of EGFR-TKIs, the survival and clinical outcomes of NSCLC patients with EGFR mutations have significantly improved. Therefore, EGFR-TKIs have been recommended as the standard first-line treatments for patients with advanced NSCLC carrying EGFR mutations [14]. Currently, three generations of EGFR-TKIs have been approved for treating NSCLC patients with different clinical presentations and specific EGFR mutations. The T790M mutation, resulting from a threonine substitution at position 790 in exon 20 of the EGFR gene by methionine, leads to drug resistance in patients receiving first- and second-generation EGFR-TKIs. It is the most common mechanism accounting for drug resistance in approximately 50-60% of these individuals [15]. With the advancement of third-generation EGFR-TKIs, particularly osimertinib, resistance to EGFR-TKIs caused by the T790M secondary mutation can be overcome. Based on findings from the FLAURA study, osimertinib is recommended as a first-line treatment for patients with advanced NSCLC presenting with activating EGFR mutations, irrespective of their T790M status [16, 17]. However, similar to patients receiving first- and second-generation EGFR-TKIs, patients receiving osimertinib also experience acquired resistance, which involves a more intricate mechanism [18–20]. The emergence of resistance to osimertinib poses a significant challenge in effectively treating NSCLC patients with sensitive EGFR mutations. Therefore, exploring the mechanisms underlying acquired resistance to EGFR-TKIs in NSCLC patients and identifying strategies to overcome this resistance present formidable challenges.

Based on our previous research on EGFR-TKI resistance, MCAM was selected as the subject of this investigation. The literature also indicates that MCAM plays a role in promoting cancer in various types of cancers. However, studies have reported an increase in MCAM levels in gefitinib-resistant PC9 cells [20]. Nevertheless, these findings were not replicated in the gefitinib-resistant PC9 cell line constructed in our laboratory. Therefore, the primary cell lines used in this study were the gefitinib- and osimertinib-resistant HCC827 cell lines. First, a significant difference in the expression level of MCAM was observed between sensitive and resistant lung adenocarcinoma cells. Additionally, after treatment with different concentrations of EGFR-TKIs for different durations, MCAM expression gradually increased, suggesting its potential involvement in lung adenocarcinoma resistance to EGFR-TKIs. The results of cell proliferation and cytotoxicity assays demonstrated that MCAM played a crucial role in conferring acquired resistance to EGFR-TKIs in lung adenocarcinoma cells. Furthermore, in vivo experiments confirmed that the overexpression of MCAM significantly attenuated the therapeutic efficacy of EGFR-TKIs. Notably, MCAM exists in both transmembrane and soluble forms. Recently, a novel monoclonal antibody called M2J-1 mAb was developed that specifically targets soluble MCAM rather than its membrane-bound counterpart. This innovative treatment strategy has produced promising outcomes by effectively inhibiting tumour angiogenesis and growth, thereby providing potential benefits for preventing the dissemination of MCAM-positive tumours, as well as thromboembolism [21, 22]. Our study also demonstrated that the combination of MCAM antibody with EGFR-TKIs partially attenuated resistance to EGFR-TKIs.

We assessed the phosphorylation status of tyrosine kinases to further elucidate the molecular mechanism underlying MCAM-mediated resistance to EGFR-TKIs. The results obtained from RTK chip and Western blot analyses revealed that MCAM overexpression led to an increase in JAK3 phosphorylation, whereas MCAM knockdown resulted in a decrease in JAK3 phosphorylation. However, to the best of our knowledge, no studies have demonstrated that MCAM may regulate the resistance of lung adenocarcinoma to EGFR-TKIs through the JAK3 signaling pathway. Our findings strongly suggest that MCAM may exert its effects through the activation of the JAK3 signalling pathway. Previous studies have shown that CJ14939, a JAK1 inhibitor, effectively reversed erlotinib resistance and significantly enhanced erlotinib-induced cell death in resistant cells [23]. We conducted further investigations to determine whether JAK3 inhibitors could elicit similar effects. By administering JAK3 inhibitors in combination with osimertinib, we examined the alterations in osimertinib resistance in lung adenocarcinoma cells. The results demonstrated that the combination of higher concentrations of JAK3 inhibitors and osimertinib exerted a synergistic effect in overcoming drug resistance in these cells. These findings further confirm the involvement of JAK3 in the development of resistance to EGFR-TKIs in EGFR-sensitive mutant lung adenocarcinoma, thereby offering a novel therapeutic strategy for addressing EGFR-TKI resistance.

Integrins, which are composed of two noncovalently linked α and β subunits, play pivotal roles in mediating cell–cell adhesion and cell–extracellular matrix interactions. Among these integrins, integrin β1 is the predominant subunit and has emerged as a critical mediator of tumour progression, exerting effects on various aspects, including cellular motility, adhesion, migration, proliferation, differentiation, and resistance to chemotherapy [24]. The upregulation of integrin β1 expression constitutes an important factor contributing to gefitinib resistance in EGFR-sensitive NSCLC [25]. In breast cancer, the activation of the downstream FAK and Src kinases by integrin β1 is implicated in trastuzumab and lapatinib resistance [26]. Studies conducted in lung cancer have also documented increased expression and activation of integrin β1 and Src in erlotinib-resistant cells, suggesting that the integrin β1/Src/Akt signalling pathway may play a crucial role in conferring erlotinib resistance to lung cancer cells [27]. Our research group also reported an increase in the expression of integrin αv, β1, β3, and other proteins in drug-resistant lung adenocarcinoma [9]. Through molecular docking, immunofluorescence staining, and coimmunoprecipitation techniques, we confirmed the binding between MCAM and integrin β1. Additionally, we found that the knockdown of integrin β1 can suppress the abnormal activation phenotype induced by MCAM. Our next step was to investigate the role of MCAM in activating JAK3 signalling through its interaction with integrin β1. Integrins are known to share a common signalling network with RTKs and support their oncogenic activity by promoting cancer cell proliferation, survival, and invasion [28]. In recent years, integrins have emerged as key players in drug resistance during targeted therapy. Consequently, drugs targeting the integrin family have become promising approaches for cancer treatment. Further research is needed to determine whether these targeted drugs can help overcome drug resistance in NSCLC patients with sensitive EGFR mutations.

Additionally, given the upregulation of MCAM at the mRNA level, we investigated its transcriptional regulation. Through the combination of a literature review and bioinformatics analysis, we identified STAT2 as a potential transcription factor involved in this process. In this study, we revealed an upregulation of STAT2 expression in cells resistant to EGFR-TKIs. The regulatory role of STAT2 was subsequently confirmed through qRT‒PCR and Western blot experiments. Furthermore, our dual-luciferase reporter assay demonstrated that STAT2 directly binds to the MCAM promoter region, thereby providing valuable insights into the intricate regulatory network governing MCAM expression. 

This study has certain limitations. First, the absence of tissue and serum samples from patients exhibiting sensitivity or resistance to EGFR-TKIs prevented us from investigating whether MCAM is also highly expressed in EGFR-TKIs-resistant patients. Therefore, future research should focus on collecting additional tissue and serum samples to validate MCAM expression levels in such patients and assess the clinical relevance of the association between MCAM expression and EGFR-TKI resistance. Second, this study did not identify the specific domain of MCAM that interacts with Integrin β1 to mediate its functional effects. To address this, we intend to construct truncated variants of MCAM to determine which domain is responsible for binding to Integrin β1, thereby elucidating the molecular mechanism underlying their interaction. Furthermore, our findings confirm the involvement of the MCAM/Integrin β1/JAK3 signaling axis in EGFR-TKI resistance. Thus, selective inhibitors targeting this pathway may represent a promising therapeutic strategy for overcoming EGFR-TKI resistance. Notably, JAK3 inhibitors have demonstrated clinical benefits in patients with T-cell acute lymphoblastic leukemia [29, 30]. While our in vitro experiments indicate that combining JAK3 inhibitors with EGFR-TKIs can partially reverse drug resistance in cell models, it remains unclear whether these agents exhibit synergistic effects in vivo or provide clinical benefits to patients with EGFR-TKI resistance. These questions will be explored in our future investigations.

## Conclusions

We found that MCAM expression was increased in EGFR-TKI-resistant lung adenocarcinoma and was involved in the process of EGFR-TKI resistance. Mechanistically, MCAM could bind to Integrin β1 to form a complex, thereby activating the JAK3 protein and the downstream AKT pathway to promote cell proliferation and resulting in the acquisition of EGFR-TKI resistance in sensitive cell lines, and MCAM is regulated by the transcription factor STAT2. It is noteworthy that MCAM is secreted in a soluble protein form, which allows for its detection in patient serum and offers potential clinical utility in predicting responses to EGFR-TKI targeted therapy. Furthermore, small molecule inhibitors or monoclonal antibodies directed against MCAM may represent novel therapeutic strategies for overcoming resistance to EGFR-TKIs. Our in vitro experiments indicate that the combination of JAK3 inhibitors and osimertinib can enhance treatment sensitivity. Nevertheless, further in vivo studies and clinical trials are required to confirm these findings and assess their feasibility in clinical practice. In conclusion, this study presents a promising new therapeutic approach for patients who develop resistance to EGFR-TKIs.

## Electronic supplementary material

Below is the link to the electronic supplementary material.

Supplementary Material 1

## Data Availability

The datasets used and/or analyzed during the current study are available from the corresponding author on reasonable request.

## Abbreviations

MCAM: Melanoma cell adhesion molecules
EGFR: Epidermal growth factor receptor
TKIs: Tyrosine kinase inhibitors
RTK: Receptor tyrosine kinase
NSCLC: Non-small cell lung cancer
si-RNA: Small interfering RNA
JAK3: Janus tyrosine kinase 3
STAT2: Signal transducer and activator of transcription 2
