# Supplementary material for: MCAM interacts with integrin β1 to promote EGFR-TKI resistance in lung adenocarcinoma through the JAK3 signalling pathway
Source: J Transl Med. 2025 Jul 25;23:831. doi: 10.1186/s12967-025-06874-9 (PMC12291482; doi:10.1186/s12967-025-06874-9)
Supplement: Supplementary file 1 — Supplementary Material 1 [file 12967_2025_6874_MOESM1_ESM.pdf]

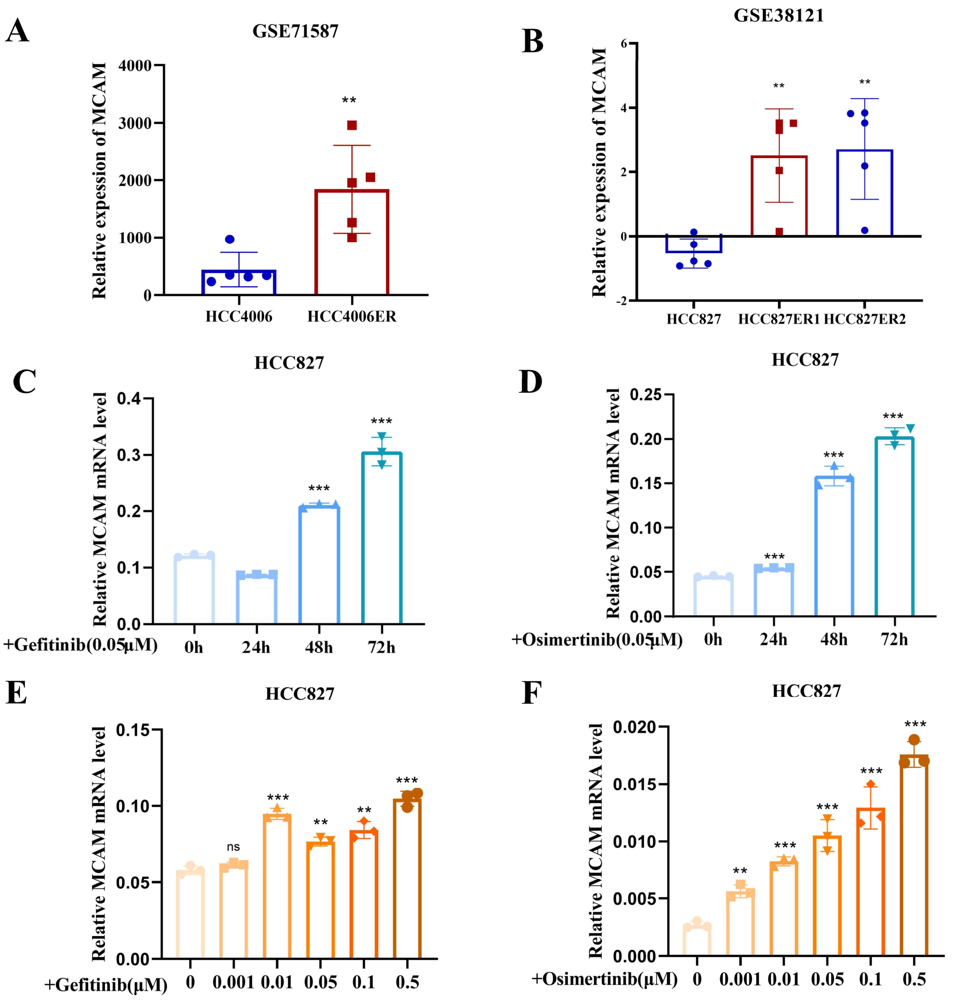

Figure S1.

(A) The GSE71587 dataset was used to detect MCAM expression in HCC4006 and HCC4006ER cells.

(B) The GSE38121 dataset was used to detect MCAM expression in HCC827 and HCC827ER cells.

(C-D) qRT–PCR was used to detect MCAM expression in HCC827 cells treated with 0.05 μmol/L gefitinib (C) or osimertinib (D).

(E-F) qRT–PCR was used to detect MCAM expression in HCC827 cells treated with different concentrations of gefitinib (E) and osimertinib (F).

**$p < 0.01$, ***$p < 0.001$ by Student's t-test.

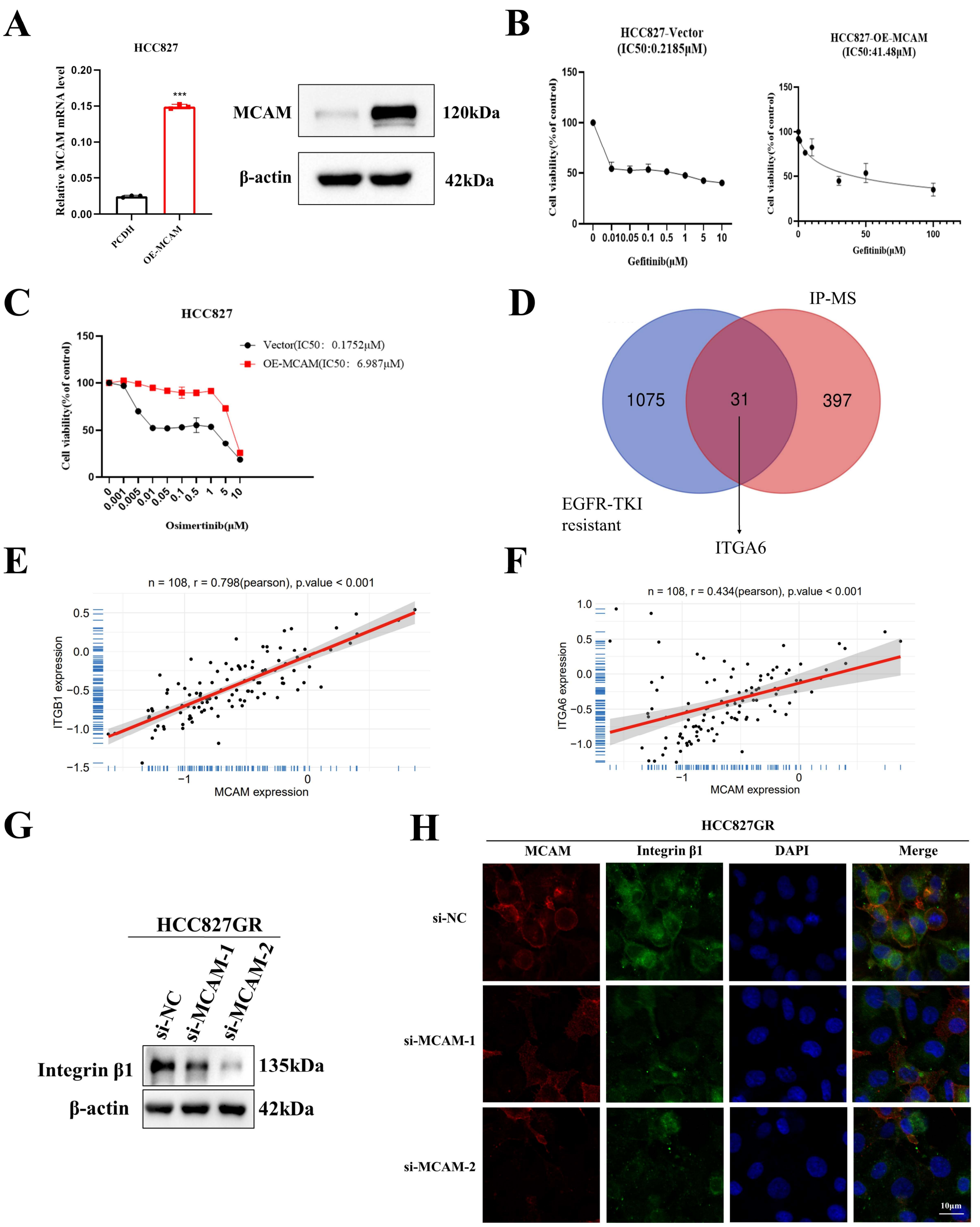

Figure S2.

(A) The mRNA and protein levels of MCAM were increased in MCAM-overexpressing cells.

(B-C) A CCK-8 assay was used to determine the IC50s of gefitinib (B) and osimertinib (C) in HCC827 cells after the overexpression of MCAM.

(D)The overlap between the differentially expressed proteins in IP_MS and the drug resistance proteins in EGFR-TKIs was determined.

(E-F) .CD146 and integrin β1 correlation analysis from the LUAD Academia protein database

(G-H) Western blotting(G) and immunofluorescence staining(H) were performed to detect Integrin β1 expression in HCC827GR cells after MCAM knockdown.scale bars = 10 μm.

**$p < 0.05$by Student's t-test.

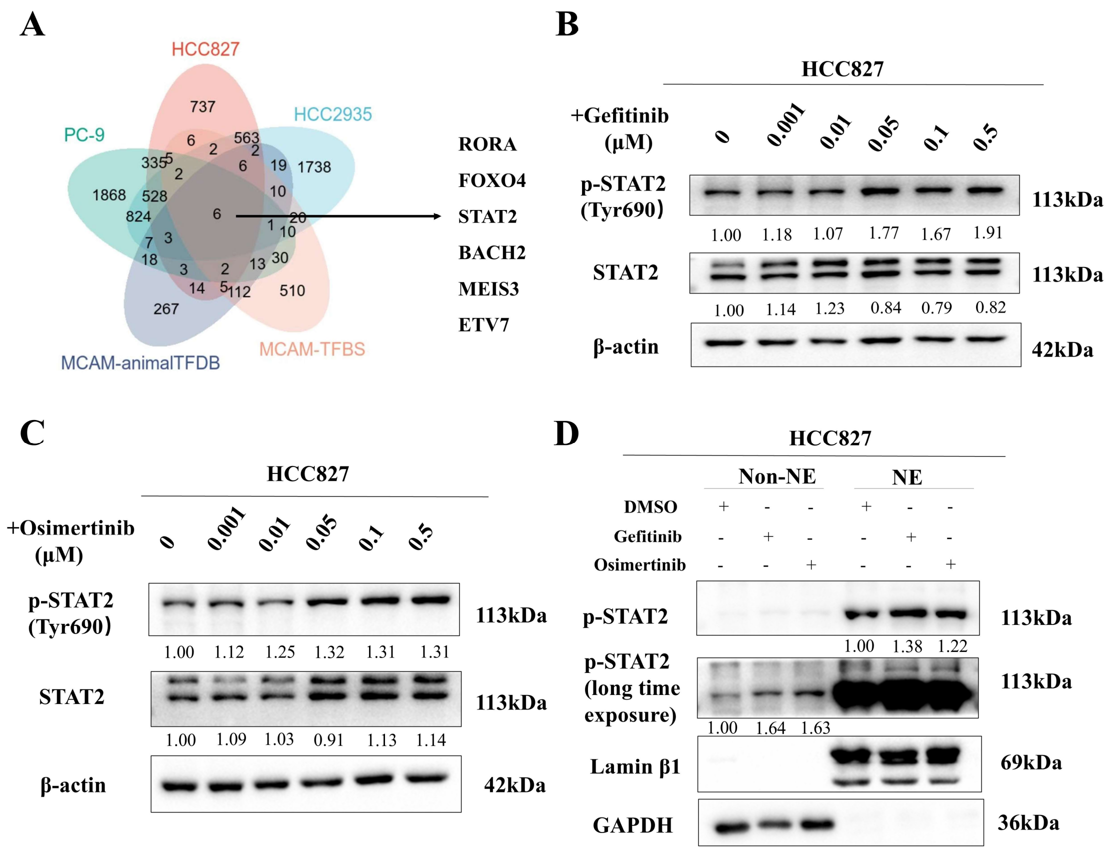

Figure S3.

(A) Venn diagram of the transcription factors predicted by the transcription factor database and upregulated in drug-resistant lung adenocarcinoma.

(B-C) Western blot analysis of STAT2 expression in HCC827 cells treated with different gefitinib (B) and osimertinib (C) concentrations.

(D) Western blot analysis of STAT2 expression in the cytoplasm and nucleus following treatment with 0.05μM gefitinib or osimertinib.

Table S1. Sequences of siRNAs.

siRNA	Sense
si-NC	5′ -TTCTCCGAACGTGTCACGT-3′
si-MCAM-1	5′ -GGUGUUGAAUCUGUCUUGUTT-3′
si-MCAM-2	5′ -GAGCGAACUUGUAGUUGAATT-3′
si-STAT2-1	5′ -CAGGAAUCCUCCUCAAUUATT-3′
si-STAT2-2	5′ -GGCCGAUUAACUACCCUAATT-3′

Table S2. Sequences of Primers for Real-time Polymerase Chain Reaction.

Gene	
MCAM Forward	AGCTCCGCGTCTACAAAGC
MCAM Reverse	CTACACAGGTAGCGACCTCC
STAT2 Forward	CUAAGAGAACUUAAACUUUTT
STAT2 Reverse	AAAGUUUAAGUUCUCUUAGCG
β-actin Forward	CACAGAGCCTCGCCTTTGCC
β-actin Reverse	ACCCATGCCCACCATCACG

TableS3 . List of signal densities of human RTK phosphorylation Antibody Array

RTKs List	HCC827		Fold Change	HCC827		Fold Change
	si-NC	si-MCAM	si-MCAM / si-NC	Vector	MCAM	MCAM/Vector
ABL1	1080.167	789.0951	0.73078	1718.769	1247.594	0.726024
ACK1	2645.833	28137.63	10.63106	33337.58	28496.66	0.854795
ALK	6773.333	4878.491	0.720291	6179.539	6671.331	1.079571
Axl	762.3333	844.4492	1.107575	684.6906	768.3382	1.12199
Blk	795.8333	688.7763	0.865647	674.4906	876.1142	1.298485
BMX	2490.667	2928.662	1.175784	2579.935	2954.278	1.145042
Btk	5233.333	6249.401	1.194116	5978.292	7014.129	1.173237
Csk	2326.5	2022.216	0.869266	2219.537	2476.239	1.115604
Dtk	641	606.8521	0.94681	576.8627	825.3552	1.43002
EGFR	1055	858.5859	0.814002	1264.468	1434.811	1.134609
EphA1	813.5	1304.655	1.603015	1027.926	1103.139	1.073098
EphA2	5794.667	5063.97	0.873924	6796.877	6927.734	1.01925
EphA3	13647.67	13096.29	0.959602	12586.88	13397.25	1.064377
EphA4	1251.667	1500.865	1.198934	1065.326	1467.665	1.377313
EphA5	4901.333	5072.486	1.034913	4930.938	5453.116	1.105877
EphA6	16295	18504.3	1.135573	17053.31	18625.94	1.092213
EphA7	5319.333	5265.971	0.98997	5111.622	5386.19	1.053704
EphA8	9554	9316.193	0.975112	8906.968	9409.015	1.056359
EphB1	11324	11270.79	0.995302	10979.82	11698.91	1.065486
EphB2	1076.833	1392.2	1.292593	1125.554	1330.164	1.181624
EphB3	1291.167	2909.756	2.252617	3844.078	1041.776	0.271198
EphB5	1998.333	1570.355	0.78594	2331.575	1826.629	0.783524
EphB6	780.1667	851.9434	1.091884	885.2892	831.4393	0.939241
ErbB2	3766.667	3460.402	0.918712	3541.642	3332.363	0.940926
ErbB3	860	858.7562	0.998555	722.0903	841.5216	1.165168
ErbB4	1185	1348.087	1.13751	1277.582	1110.614	0.869412
FAK	8964.167	6763.769	0.754561	4637.244	5178.461	1.116686
FER	2307.5	9364.053	4.05677	7814.766	8283.451	1.059967
FGFR1	112584.7	126812.2	1.12637	137702.1	162134.3	1.177426
FGFR2	5120.167	5585.151	1.090797	5539.695	6715.137	1.212147
FGFR2 (α	1387.5	1500.183	1.081155	1897.835	2084.422	1.098264

isoform)						
Fgr	1632.667	2161.368	1.323628	2192.661	1808.724	0.824979
FRK	6599.833	6375.439	0.966005	6490.232	6753.38	1.040539
Fyn	838.8333	791.8202	0.944021	1027.764	867.0749	0.843803
Hck	6817.5	6966.962	1.02192	6488.775	7208.125	1.110844
HGFR	532.6667	434.4877	0.816029	617.3386	862.2076	1.396011
IGF-I R	334.6667	721.1372	2.151352	695.8619	667.1679	0.958824
Insulin R	6456	5898.541	0.913666	5670.837	5928.894	1.045498
Itk	7185.5	7842.75	1.091456	7726.852	8811.553	1.140363
JAK1	5976.333	5152.026	0.862094	6208.034	6074.739	0.978532
JAK2	866.8333	1176.404	1.356717	911.1938	968.7668	1.063115
**JAK3**	**5158.5**	**3584.907**	**0.695011**	**5680.228**	**6828.476**	**1.202113**
LCK	1923.167	1773.888	0.922419	1880.673	2273.725	1.208884
LTK	746.6667	695.0781	0.931001	773.8995	806.4075	1.041951
Lyn	9226.667	9779.976	1.059962	9543.573	10321.11	1.081464
MATK	12427	13301.86	1.070395	13123.91	13403.33	1.021289
M-CSFR	1610	1570.526	0.975497	1491.618	1551.452	1.040087
MUSK	14136	14722.17	1.041463	14338.84	15988.39	1.115033
NGFR	2580.167	2274.971	0.881761	3892.326	3356.873	0.862469
PDGFR-α	2815.833	3598.532	1.277865	3307.692	3937.125	1.190236
PDGFR-β	253.1667	431.2516	1.700662	382.4164	239.0193	0.626001
PYK2	1082.667	1231.758	1.137581	899.051	1048.73	1.1663
RET	1088.167	1344.51	1.235358	2097.3	1548.497	0.738454
ROR1	343	635.2956	1.849697	709.7856	565.6499	0.797216
ROR2	1192.667	1236.697	1.036887	969.8029	1685.651	1.737377
ROS	3219	3171.368	0.985208	2742.648	3055.101	1.113882
RYK	1136.333	1342.637	1.181392	1334.896	742.7849	0.556769
SCFR	717.1667	617.5823	0.861335	634.6623	1072.197	1.688313
SRMS	11087.67	13119.96	1.183277	12233.12	12644.55	1.03363
SYK	15051.5	15155.97	1.006941	14157.18	16016.2	1.131304
Tec	244.1667	383.2212	1.567184	328.1787	417.7187	1.27201
Tie-1	2336.333	3810.752	1.630812	1970.529	1865.915	0.946937
Tie-2	557.6667	873.0631	1.564552	693.2715	934.1741	1.346986
TNK1	2964.5	3534.833	1.192323	3514.928	3487.421	0.992176
TRKB	7750.167	7189.06	0.92761	8592.875	9215.018	1.072394
TXK	4156.833	2408.503	0.579509	5750.818	5668.146	0.985627

Tyk2	9887.167	10090.81	1.020595	9756.8	9112.457	0.933966
TYRO10	708.1667	833.0378	1.176081	840.28	678.6408	0.807865
VEGFR2	803.6667	903.0395	1.123496	813.5659	850.9085	1.045844
VEGFR3	1408.5	1253.73	0.890195	1726.379	2105.455	1.219452
ZAP70	6733.5	5776.932	0.85796	5847.312	6866.719	1.174308
